# Early Maladaptive Schemas Mediate the Relationship Between Childhood Trauma and Interpersonal Problems in Eating Disorders

**DOI:** 10.1002/cpp.70052

**Published:** 2025-03-16

**Authors:** Matteo Aloi, Marianna Rania, Elvira Anna Carbone, Renato de Filippis, Daria Quirino, Ettore D'Onofrio, Cristina Segura‐Garcia

**Affiliations:** ^1^ Department of Clinical and Experimental Medicine University of Messina Messina Italy; ^2^ Outpatient Unit for Clinical Research and Treatment of Eating Disorders University Hospital Renato Dulbecco Catanzaro Italy; ^3^ Department of Health Sciences University “Magna Graecia” of Catanzaro Catanzaro Italy; ^4^ Department of Medical and Surgical Sciences University “Magna Graecia” of Catanzaro Catanzaro Italy

**Keywords:** childhood adversities, path analysis, psychotherapy, schema therapy, social functioning, targeted intervention

## Abstract

Recent research highlights the role of childhood trauma in the development of eating disorders (EDs), suggesting that adverse experiences can disrupt emotional and cognitive development, leading to early maladaptive schemas (EMSs) and interpersonal problems. EMSs are psychological structures encompassing cognition, emotions, images, and bodily sensations. These EMSs are hypothesized to mediate the relationship between childhood trauma and ED pathology. This study aimed to extend the investigation of how childhood trauma leads to interpersonal difficulties, mediated by EMSs.

This cross‐sectional study recruited 321 patients with EDs: *n* = 77 Anorexia Nervosa–Restricting (AN‐R), *n* = 26 AN–Binge/Purge (AN‐BP), *n* = 94 Bulimia Nervosa (BN), and *n* = 124 Binge Eating Disorder (BED). They completed the Eating Disorder Examination (EDE), Inventory of Interpersonal Problems (IIP‐32), Young Schema Questionnaire (YSQ‐S3), and Childhood Trauma Questionnaire (CTQ‐SF). Path analyses were conducted to examine associations between childhood trauma, EMSs, and interpersonal problems.

Results indicated that patients with BED reported the highest levels of sexual abuse. Those with BN exhibited higher scores across all EMS domains. Positive correlations were found between childhood adversities, EMS domains, and interpersonal problems, except for the relationship between sexual abuse and schema domains. Mediation analyses revealed significant indirect effects of emotional abuse on interpersonal problems through disconnection and rejection domain.

This study consolidates the notion that EMSs mediate the relationship between childhood trauma and interpersonal problems in ED patients, highlighting the importance of addressing early trauma and EMSs to improve therapeutic outcomes. Future research should focus on longitudinal analyses to better understand the temporal development of these relationships.

**Summary**

- Childhood trauma impacts ED development through EMSs and interpersonal issues.
- Schema‐focused interventions may improve both interpersonal and ED symptoms.
- Addressing interpersonal problems can enhance treatment outcomes for ED patients.

## 1 Introduction

Eating disorders (EDs) represent a significant public health concern (Himmerich et al. 2024) characterized by abnormal eating behaviours and worries about body weight and shape (Fairburn et al. 2009). These disorders often lead to severe physical and psychological complications. While the aetiology of EDs is multifaceted, encompassing biological, psychological, and social factors (Aloi et al. 2024; Hay and Mitchison 2014), the role of childhood trauma has garnered substantial attention in recent research. Childhood trauma, defined as adverse experiences during formative years, such as physical, emotional, or sexual abuse, as well as neglect, has been consistently linked to the development of various psychopathologies, including EDs (Carvalho Silva et al. 2024; Pignatelli et al. 2017). Childhood adversities can have a profound and lasting impact on an individual's psychological development. Traumatic experiences during childhood disrupt normal emotional and cognitive development, leading to a range of maladaptive outcomes (Eielsen et al. 2024). These adverse experiences are known to impair the ability to form and maintain healthy interpersonal relationships, as they often result in deep‐seated feelings of mistrust, fear, and insecurity. Persons who have experienced childhood trauma frequently exhibit heightened sensitivity to perceived threats and rejection, which can significantly affect their social interactions and relationships (Cloitre et al. 2009).

In this framework, schemas are fundamental psychological constructs that represent deeply ingrained patterns of thought, emotion and behaviour, shaped by early life experiences. In Young's schema therapy model, early maladaptive schemas (EMSs) are defined as pervasive psychological structures that encompass not only cognitive content but also emotional states, bodily sensations, and mental images. These schemas are theorized to emerge when core emotional needs—such as safety, autonomy, and connection—are unmet during childhood (Young et al. 2003).

Parallel conceptualizations of schemas exist within other theoretical frameworks, which can provide additional insights. For example, internal working models from attachment theory describe mental representations of self and others that guide interpersonal interactions, often shaped by early caregiver relationships (Bowlby 1988). Similarly, cognitive models, such as those proposed by Beck (1964), conceptualize schemas as dysfunctional beliefs or cognitive structures that influence information processing and perpetuate maladaptive behaviours. From an object relations perspective, schemas can be understood as internalized object relations, where early interactions with caregivers shape expectations and patterns of relating to others (Kernberg 1976).

In the context of childhood trauma, EMSs are enduring psychological frameworks shaped by unmet emotional needs and adverse experiences, which assign meaning to these events. These schemas, once formed, become stable representations about the self and others, influencing thoughts, emotions, and behaviours in pervasive ways (Joshua et al. 2024; Maher et al. 2023). By situating EMSs within these broader conceptual frameworks, we can better understand their role in psychological functioning and their relevance to EDs.

Further, interpersonal problems are a hallmark feature of many individuals with EDs. These problems often manifest as difficulties in establishing and maintaining close relationships, conflicts with family members and peers, social withdrawal, and a pervasive sense of isolation (Monteleone et al. 2023; Sahota et al. 2024). The link between childhood trauma and interpersonal problems in EDs could be partially explained by the EMSs that develop in response to trauma. For example, an individual who feels unworthy of love may struggle with forming intimate relationships and may engage in disordered eating behaviours as a means of coping with feelings of rejection and inadequacy. While schemas themselves are not coping mechanisms, they can inform the coping styles or strategies individuals develop to manage distress. Maladaptive interpersonal patterns, often conceptualized as ‘interpersonal problems,’ can serve as coping mechanisms aimed at dealing with perceived threats or unmet needs. These patterns align with Young's schema therapy model, in which coping styles—such as overcompensation, avoidance, and surrender—function as strategies to manage the distress associated with EMSs (Young et al. 2003).

In this vein, these parallel models highlight the interplay between early experiences and the development of maladaptive cognitive and emotional patterns, providing a comprehensive foundation for examining the mediational role of EMSs in the relationship between childhood trauma and interpersonal problems in ED populations. Trauma‐induced negative schemas can perpetuate a cycle of maladaptive thoughts and behaviours, leading to chronic interpersonal difficulties. For instance, a person with a schema of mistrust may constantly anticipate betrayal or rejection in relationships, leading them to engage in defensive or avoidant behaviours that, in turn, exacerbate interpersonal problems. These maladaptive schemas also contribute to the maintenance of disordered eating behaviours, as individuals may use food and body control as a means of managing their emotional distress and compensating for perceived interpersonal failures.

Prior research has demonstrated that EMSs, particularly Disconnection and Rejection (DR) schema domain, mediate the impact of childhood trauma in several psychiatric disorders: psychological distress (Azadfar et al. 2025), depressive symptoms (Li et al. 2020), substance use (Olave et al. 2024) and psychotic experiences (Boyda et al. 2018). In the context of EDs, Meneguzzo et al. (2021) underscored the role of EMSs in the complex interplay between trauma, personality traits, and clinical severity. Similarly, Fasolato et al. (2024) investigated the mediational role of EMSs in linking severe childhood trauma to eating disorder symptoms, providing further evidence of their significance in this relationship.

These findings provide a strong theoretical foundation for examining the mediating role of schemas in our study. By focusing on the role of EMSs in linking childhood trauma and interpersonal difficulties, this research extends existing evidence and offers new insights into transdiagnostic mechanisms relevant to EDs.

Based on the above, the present research has twofold primary aims: (1) to explore potential differences in childhood trauma, EMSs, and interpersonal problems across ED and (2) their relationship; (3) to evaluate the mediating role of EMSs in the relationship between childhood trauma and interpersonal problems. These objectives aim to provide a deeper understanding of how early traumatic experiences contribute to the psychological and interpersonal challenges observed in ED populations. In line with these aims and the previous studies, the study hypothesizes the following: (1) childhood trauma, EMSs, and interpersonal problems show significant variability among individuals with EDs; (2) higher levels of childhood adversities (e.g., emotional abuse, physical abuse, neglect) are positively associated with elevated scores across all EMS domains and greater interpersonal problems; and (3) EMSs, particularly in the DR schema domain, mediate the relationship between emotional abuse and interpersonal difficulties, highlighting the central role of these schemas in linking early trauma to interpersonal dysfunction in EDs.

## 2 Methods

### 2.1 Participants

Participants for this cross‐sectional study were recruited from the Outpatient Unit for Clinical Research and Treatment of Eating Disorders at the University Hospital ‘Renato Dulbecco’ in Catanzaro, Italy, between December 2018 and June 2024. Eligible participants were consecutively enrolled during their initial visit, following an explanation of the study's aim and procedures by the research team. Inclusion criteria required participants to be aged between 14 and 65, diagnosed with an eating disorder (ED) according to DSM‐5 criteria (American Psychiatric Association 2013), willing to participate, and able to provide valid informed consent. Exclusion criteria included severe psychiatric comorbidities (e.g., neurodevelopmental disorders, disorders in the schizophrenia spectrum, bipolar disorders, neurocognitive disorders), neurological or medical conditions (e.g., diabetes), active substance dependence or abuse (within ≤ 6 months), and other medical conditions or treatments that could influence eating behaviours.

Each participant underwent a diagnostic interview conducted by experienced psychiatrists using the Structured Clinical Interview for DSM‐5 (SCID‐5‐CV) (First et al. 2016) and the Eating Disorder Examination (EDE 17.0D) (Calugi et al. 2015). Subsequently, they completed self‐report questionnaires assessing psychological aspects such as childhood trauma, maladaptive schemas and interpersonal problems.

Of the 336 patients initially approached, 15 were excluded: three were active substance users (0.9%), five dropped out before assessment completion (1.5%), three had an intellectual disability (0.9%), and four exhibited psychotic symptoms (1.2%). The final sample consisted of 321 patients: *n* = 77 Anorexia Nervosa – Restricting (AN‐R), *n* = 26 Anorexia Nervosa – Binge/Purge (AN‐BP), *n* = 94 Bulimia Nervosa (BN), and *n* = 124 Binge Eating Disorder (BED) with a dropout rate of 4.5%.

Only those who agreed to participate, provided informed consent, and completed the evaluation were included in the analysis. There were no missing data in the participants' socio‐demographic information or assessments. The study adhered to the ethical principles outlined in the Helsinki Declaration (World Medical Association 2013) and received approval from the Ethical Committee of ‘Regione Calabria, Sezione Area Centro’ (identifier: Prot. 66/15.03.2018). Written informed consent was obtained from all participants, and for minors, consent was acquired from their parents or legal guardians after providing detailed information.

### 2.2 Measures

- **Eating Disorder Examination (EDE):** This clinical interview evaluates the presence and severity of eating psychopathology through four dimensions: Eating Restraint, Eating Concern, Weight Concern, and Shape Concern, culminating in a global EDE score (Calugi et al. 2015). This semi‐structured interview covers ED‐related behaviours and psychopathology over the past three months, addressing behavioural symptoms such as binge eating, self‐induced vomiting, diuretic and laxative misuse, excessive exercise, and food restriction. Higher scores indicate greater severity of psychopathology. Original validation alphas range from 0.65 to 0.84 across subscales (Fairburn et al. 2008) and, in our sample, the internal consistency reliability (McDonald's ω) indexes were: Restraint = 0.78; Eating Concern = 0.75; Weight Concern = 0.78; Shape Concern = 0.83; Global score = 0.84.
- **Inventory of Interpersonal Problems 32‐item version (IIP‐32):** This questionnaire assesses interpersonal functioning by exploring interpersonal difficulties (Horowitz et al. 2000), conceptualized in terms of dominance and affiliation, across eight sub‐scales: Overly Accommodating/Exploitable that reflects a tendency to prioritize others' needs at the expense of one's own, often resulting in being taken advantage of or feeling unappreciated; Vindictive/Self‐centred that represents difficulty in compromising, a lack of empathy, and a tendency toward controlling or hostile behaviour in interpersonal relationships; Intrusive/Needy that characterizes excessive dependence on others for emotional support, difficulty respecting boundaries, and a tendency to seek constant reassurance; Socially Inhibited/Avoidant that reflects discomfort in social situations, avoidance of interpersonal interactions, and a fear of rejection or criticism; Non‐assertive that represents difficulty in expressing personal needs or standing up for oneself, often leading to passive or submissive behaviour in relationships; Cold/Distant that characterizes emotional detachment, difficulty forming close relationships, and a lack of warmth or empathy toward others; Self‐sacrificing/Overly nurturant that reflects a tendency to excessively care for others, often neglecting personal needs and boundaries to support others; and Domineering/Controlling that represents a tendency to dominate or control others in interpersonal relationships, often at the expense of collaboration or mutual respect. Original validation alphas range from 0.70 to 0.87 (Horowitz et al. 2000) while, in the present study, omega coefficients were: Overly Accommodating/Exploitable (ω = 0.73), Vindictive/Self‐centred (ω = 0.73), Intrusive/Needy (ω = 0.82), Socially Inhibited/Avoidant (ω = 0.82), Non‐assertive (ω = 0.80), Cold/Distant (ω = 0.75), Self‐sacrificing/Overly nurturant (ω = 0.76) and Domineering/Controlling (ω = 0.78). The total score, derived from the sum of these subscales, had an ω of 0.84 (Lo Coco et al. 2018).
- **Young Schema Questionnaire – Short Form 3 (YSQ‐S3):** This self‐report inventory contains 90 items rated on a six‐point scale (from ‘completely untrue of me’ to ‘describes me perfectly’). According to Bach et al. (2018), the 90 items are grouped into 18 Early Maladaptive Schemas (EMSs) clustered into four domains: 1) Disconnection & Rejection (DR) represents schemas that stem from unmet needs for safety, security, love, and belonging, 2) Impaired Autonomy & Performance (IA & P) includes schemas that arise from an inability to develop independence and confidence, 3) Excessive Responsibility & Standards (ER & S) comprises schemas that are characterized by rigid rules, perfectionism, and the need to meet excessively high standards, and 4) Impaired Limits (IL) reflects difficulties in setting boundaries or adhering to appropriate limits. Original validation omega ranges from 0.70 to 0.89 across subscales (Aloi, Rania, Caroleo, et al. 2020) while in our study, omega coefficients ranged from 0.76 (ER & S) to 0.84 (IA & P).
- **Childhood Trauma Questionnaire Short‐Form (CTQ‐SF):** This self‐administered test comprises 28 Likert‐scale items. It evaluates childhood maltreatment across five subscales and each subscale measures distinct types of adverse childhood experiences: emotional abuse, physical abuse, sexual abuse, emotional neglect and physical neglect (Innamorati et al. 2016). Original validation alphas range from 0.83 to 0.92 (Bernstein et al. 2003) and in our study, omega coefficients were as follows: physical abuse 0.83; emotional abuse 0.87; sexual abuse 0.85; emotional neglect 0.90; and physical neglect 0.85.

### 2.3 Statistical Analysis

Patients were initially compared across sub‐samples (AN‐R, AN/BP, BN, and BED) for socio‐demographic characteristics and study variables using ANOVA, followed by Bonferroni post hoc tests to identify group‐specific differences. While this step allowed us to explore variations among subtypes, the subsequent correlational and mediational analyses were conducted on the combined ED sample. This approach was guided by the transdiagnostic theory of eating disorders (Fairburn et al. 2003), which posits a shared psychopathological core across ED subtypes. The theory suggests that despite subtype‐specific features, EDs share common underlying mechanisms, justifying the analysis of associations between the study variables and interpersonal problems within a merged sample.

According to the research question, the magnitude of association between variables (CTQ subscales, YSQ domains, IIP total score) and the mediational path was examined. We tested if the variables of interest (the dependent, the independent, and the mediator variables) had a linear relationship, which could be checked with a correlation matrix and scatterplots.

Mediation analyses were conducted by using JASP software (JASP Team 2024). A multiple mediator's path model was run with CTQ subscores as independent variables, interpersonal difficulties as dependent variables and YSQ domains as mediators. Confounding variables, particularly those found to be significant in the ANOVA comparisons, were included in the analysis to control for their potential effects. The statistical significances of the mediating and indirect effects were assessed using bootstrapped procedure (namely, running percentile‐based confidence interval of 5000 bootstrap) and the maximum likelihood robust (MLR) estimator (Preacher and Hayes 2008).

## 3 Results

Table 1 summarizes the socio‐demographic features and clinical data for the total sample and each group. In the overall sample, most participants were female, had completed high school, and were single. Significant differences emerged in BMI and age across groups. The BED group tends to have a higher age and BMI compared to the other groups. The BN group is associated with a higher BMI and age than the AN‐R and AN‐BP. Finally, AN‐BP has a higher average BMI than AN‐R but there is no significant difference in age between the anorexia subtypes.

**TABLE 1:** Socio‐demographic and clinical data of the sample.

		Total sample		AN‐R		AN‐BP		BN		BED				
		*N* = 321		*n =* 77		*n =* 26		*n =* 94		*n =* 124		χ^2^/F	*p*	Post hoc
		Mean	SD	Mean	SD	Mean	SD	Mean	SD	Mean	SD			
Age^a^		28.2	13.7	18.6	6.6	24.1	10.1	22.9	8.2	38.5	13.8	63.824	**<0.001**	4 > all; 3 > 1
Sex^b^	Female	292	91.0	72	93.5	23	88.5	87	92.6	110	88.7	1.859	0.602	
	Male	29	9.0	5	6.5	3	11.5	7	7.4	14	11.3			
BMI^a^		27.6	11.4	17.1	2.3	19.0	1.9	23.8	5.3	38.8	8.6	209.508	**<0.001**	4 > all; 3 > 1,2;2 > 1
Civil status^b^	Married	89	27.7	4	5.2	4	15.4	16	17.0	65	52.4	80.876	**<0.001**	
	Single	223	69.5	73	94.8	22	84.6	77	81.9	51	41.1			
	Divorced	9	2.8	0	0	0	0	1	1.1	8	6.5			
Education^b^	Elementary	4	1.2	2	2.6	0	0	1	1.1	1	0.8	33.659	**<0.001**	
	Middle school	125	38.9	45	58.4	14	53.8	36	38.3	30	24.2			
	High school	151	47.0	28	36.4	9	34.6	46	48.9	68	54.8			
	Master	41	12.8	2	2.6	3	11.5	11	11.7	25	20.2			
YSQ‐S3^a^	Disconnection and rejection	3.0	1.0	2.8	1.1	3.3	1.2	3.4	1.1	2.8	0.9	7.449	**<0.001**	3 > 1,4
	Impaired autonomy and performance	2.7	1.0	2.4	0.9	3.0	1.1	3.1	1.0	2.6	0.9	9.265	**<0.001**	2,3 > 1; 3 > 4
	Excessive responsibilities and standards	3.5	0.9	3.4	0.9	3.9	1.1	3.7	1.0	3.4	0.8	4.917	0.**002**	2,3 > 4
	Impaired limits	3.0	0.9	2.7	0.8	2.9	1.0	3.1	0.9	3.1	1.0	3.887	0.**009**	3,4 > 1
CTQ‐SF^a^	Emotional abuse	9.6	4.6	8.7	4.7	9.5	4.4	10.5	4.9	9.4	4.2	1.874	0.135	
	Physical abuse	6.1	2.4	5.5	1.1	6.9	3.1	6.3	2.6	6.2	2.6	2.483	0.061	
	Sexual abuse	6.5	3.8	5.3	1.6	7.2	3.9	6.9	4.7	6.9	4.0	2.689	0.**047**	4 > 1
	Emotional neglect	10.6	4.8	9.8	5.0	10.5	5.2	11.4	4.6	10.6	4.8	1.360	0.256	
	Physical neglect	6.3	2.0	6.0	1.7	6.5	1.9	6.2	1.7	6.6	2.3	1.114	0.344	
IIP‐32^a^	Domineering/controlling	3.2	3.5	3.1	3.6	1.4	1.4	3.5	3.7	3.4	3.4	1.268	0.287	
	Vindictive/self‐centred	2.9	3.5	2.6	3.1	2.9	2.8	3.4	3.9	2.5	3.5	0.729	0.536	
	Cold/Distant	4.7	3.8	4.6	3.8	7.6	2.2	5.4	3.8	3.4	3.6	5.476	0.**001**	2 > 1; 2,3 > 4
	Socially inhibited/avoidant	7.5	5.0	7.7	5.2	11.7	3.9	8.9	4.6	5.3	4.5	8.611	0.**000**	2,3 > 4
	Non‐assertive	6.7	3.9	5.9	4.0	8.2	3.7	7.9	3.6	6.0	4.0	3.316	0.**021**	
	Overly accommodating/exploitable	7.9	3.8	7.0	4.2	9.1	4.3	8.9	3.1	7.4	3.8	2.850	0.**039**	
	Self‐sacrificing/overly nurturant	8.2	3.5	7.3	3.4	9.5	3.6	8.9	3.3	7.8	3.4	2.461	0.065	
	Intrusive/needy	4.6	3.9	3.1	3.3	3.0	3.1	4.7	3.8	6.0	4.0	5.972	0.**001**	4 > 1
	Total score	45.7	18.3	41.4	20.0	53.4	12.9	51.6	15.7	41.9	18.6	4.446	0.**005**	3 > 1,4

*Note:* 1: AN‐R; 2: AN‐BP; 3: BN; 4: BED. Significant results are in bold.

Abbreviations: BMI: body mass index; CTQ‐SF: Childhood Trauma Questionnaire – Short Form; IIP‐32: Inventory of Interpersonal Problems – 32; SD: Standard Deviation; YSQ‐S3: Young Schema Questionnaire – Short Form 3.

^a^ Data are presented as means (SD).

^b^ Data are presented as frequencies (%).

No differences were found in the mean scores of the variables of interest across the four CTQ subscales, except on the sexual abuse subscale, where the BED group reported a higher score than the AN‐R group. On the YSQ‐S3, the BN group reported higher scores across all domains compared to the BED group (in the DR, IA&P, and ER&S domains) and the AN‐R group (in the DR and IL domains). The AN‐BP group showed higher scores than the AN‐R group (in the IA&P domain) and the BED group (in the ER&S domain). Additionally, the BED group scored higher than the AN‐R group in the IL domain.

In terms of interpersonal problems, the BN group had higher total score than the AN‐R and BED groups. Notably, the BED group showed higher scores in the Intrusive/Needy domain than the AN‐R group, and the AN‐BP group displayed higher scores in the Cold/Distant subscale than the AN‐R group. Lastly, the AN‐BP and BN groups reported higher scores than the BED group in the Cold/Distant and Socially Inhibited/Avoidant subscales.

The correlation matrix (Table 2) and the scatterplots (Table S1) indicated significant linear associations between the variables of interest. Specifically, significant positive associations were found between interpersonal problems and schema domains, as well as between interpersonal problems and childhood adversities, except for physical abuse. Schema domains also showed a strong positive relationship with childhood adversities, except for sexual abuse.

**TABLE 2:** Correlation matrix depicting the relationships among the variables of interest.

	DR	IA & P	ER & S	IL	EA	PA	SA	EN	PN	IIP
DR	—									
IA & P	0.**797** ***	—								
ER & S	0.**628** ***	0.**627** ***	—							
IL	0.**565** ***	0.**596** ***	0.**429** **	—						
EA	0.**380** ***	0.**354** ***	0.330***	0.**274** ***	—					
PA	0.**130** *	0.**147** *	0.076	0.120	0.**456** ***	—				
SA	−0.007	0.067	0.083	0.063	0.**193** **	0.**422** **	—			
EN	0.**260** ***	0.**282** ***	0.**140** ^a^	0.**233** ***	0.**616** ***	0.**237** ***	0.**145** *	—		
PN	0.**179** **	0.**246** ***	0.111	0.**226** ***	0.**461** ***	0.**343** ***	0.**260** ***	0.**586** ***	—	
IIP	0.**659** ***	0.**644** ***	0.**407** *	0.**523** ***	0.**280** ***	0.076	**−0.164** *	0.**281** ***	0.**182** *	—

*Note:* Significant results are in bold.

Abbreviations: DR: disconnection and rejection; EA: emotional abuse; EN: emotional neglect; ER & S: excessive responsibilities and standards; IA & P: impaired autonomy and performance; IIP: inventory of interpersonal problems total score; IL: impaired limits; PA: physical abuse; PN: physical neglect; SA: sexual abuse.

** *p* < 0.01.

* *p* < 0.05.

*** *p* < 0.001.

**FIGURE 1:**
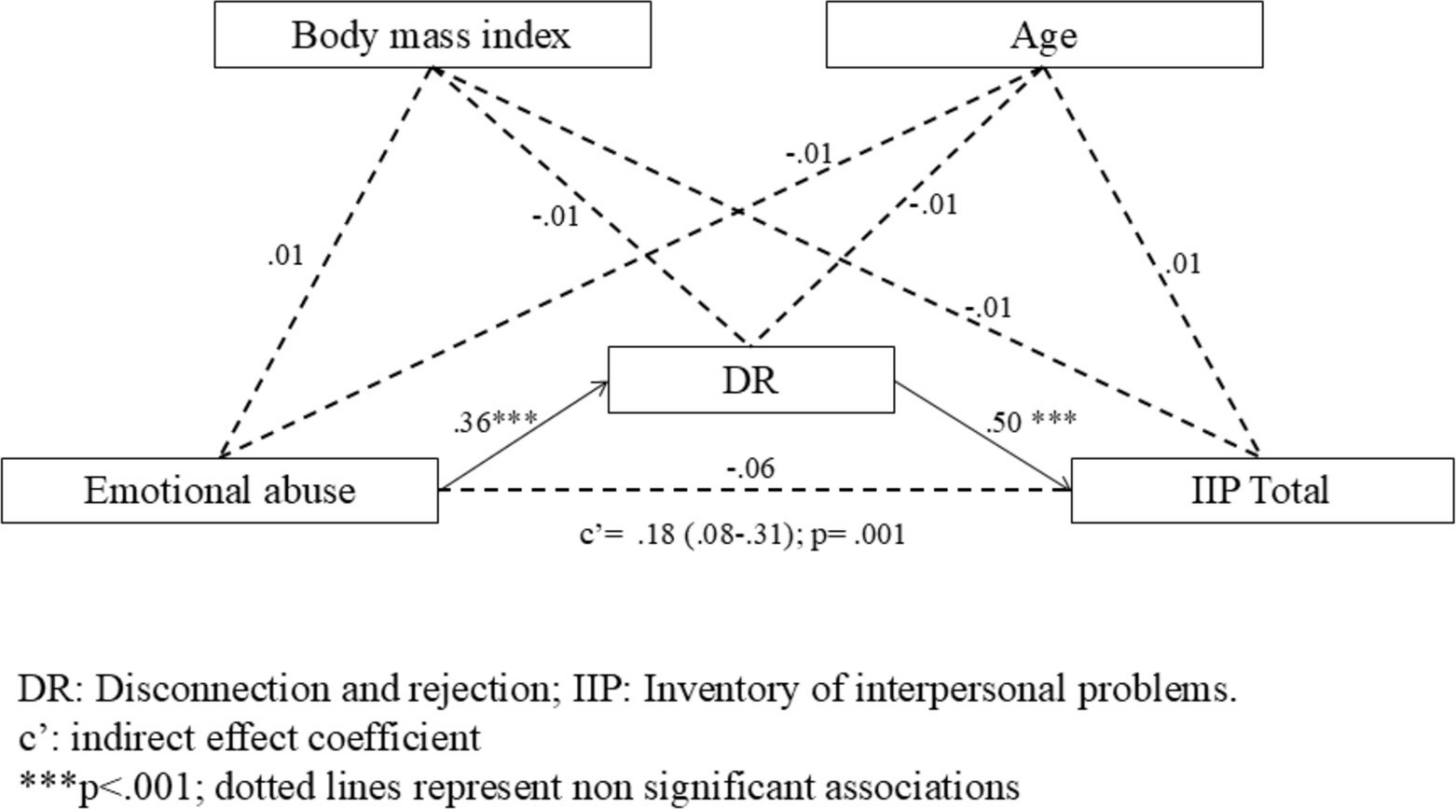
Graphical representation of the mediation analysis examining the relationship between emotional abuse (independent variable), IIP total score (dependent variable), and EMS domains (mediator), with age and BMI included as confounding variables. This figure highlights the only significant indirect association identified in the mediation analysis.

All path coefficients from the mediation analysis are displayed in Table 3. Significant paths were identified from emotional abuse to all YSQ‐S3 schema domains and from the DR, IA&P, and IL schema domains to the IIP total score. The indirect effect of emotional abuse on the IIP total score via DR (β = 0.18; *p* = 0.001; CI 95% [0.10, 0.30]) was confirmed (Figure 1).

**TABLE 3:** Parameter estimates of the path diagram model.

Direct effects									
								95% confidence interval	
					β	SE	p	Lower	Upper
EA			→	IIP Total	−0.058	0.089	0.515	−0.263	0.153
PA			→	IIP Total	−0.199	0.093	0.056	−0.460	0.060
SA			→	IIP Total	−0.019	0.072	0.795	−0.189	0.145
EN			→	IIP Total	0.054	0.088	0.541	−0.185	0.281
PN			→	IIP Total	−0.102	0.078	0.190	−0.268	0.079

Path coefficients									
DR			→	IIP Total	0.494	0.105	**<0.001**	0.264	0.703
IA & P			→	IIP Total	0.211	0.104	0.**042**	−0.051	0.474
ER & S			→	IIP Total	0.021	0.076	0.782	−0.156	0.189
IL			→	IIP Total	0.193	0.070	0.**006**	0.044	0.334
EA			→	IIP Total	−0.058	0.089	0.515	−0.263	0.153
PA			→	IIP Total	−0.199	0.093	0.056	−0.460	0.060
SA			→	IIP Total	−0.019	0.072	0.795	−0.189	0.145
EN			→	IIP Total	0.054	0.088	0.541	−0.185	0.281
PN			→	IIP Total	−0.102	0.078	0.190	−0.268	0.079
EA			→	DR	0.357	0.079	**<0.001**	0.198	0.503
PA			→	DR	0.024	0.073	0.740	−0.109	0.214
SA			→	DR	−0.064	0.070	0.361	−0.241	0.110
EN			→	DR	0.059	0.084	0.482	−0.120	0.229
PN			→	DR	0.018	0.078	0.817	−0.130	0.180
EA			→	IA & P	0.247	0.083	0.**003**	0.077	0.420
PA			→	IA & P	−0.014	0.076	0.853	−0.196	0.222
SA			→	IA & P	−0.063	0.073	0.386	−0.226	0.102
EN			→	IA & P	0.076	0.088	0.387	−0.118	0.258
PN			→	IA & P	0.125	0.081	0.123	−0.046	0.295
EA			→	ER & S	0.475	0.079	**<0.001**	0.309	0.631
PA			→	ER & S	−0.097	0.074	0.187	−0.236	0.064
SA			→	ER & S	0.058	0.071	0.412	−0.091	0.216
EN			→	ER & S	−0.068	0.085	0.421	−0.232	0.093
PN			→	ER & S	−0.030	0.078	0.697	−0.182	0.133
EA			→	IL	0.169	0.086	0.**048**	−0.036	0.367
PA			→	IL	−0.011	0.079	0.894	−0.198	0.185
SA			→	IL	−0.083	0.076	0.269	−0.238	0.059
EN			→	IL	0.016	0.091	0.861	−0.183	0.208
PN			→	IL	0.165	0.083	0.056	−0.006	0.332
Age			→	EA	−0.007	0.006	0.272	−0.018	0.005
BMI			→	EA	0.012	0.007	0.101	−0.002	0.027
Age			→	PA	0.010	0.006	0.081	−0.002	0.027
BMI			→	PA	0.002	0.007	0.826	−0.012	0.016
Age			→	SA	0.007	0.006	0.272	−0.006	0.019
BMI			→	SA	0.004	0.008	0.624	−0.010	0.022
Age			→	EN	−0.004	0.006	0.466	−0.015	0.007
BMI			→	EN	0.010	0.007	0.181	−0.003	0.023
Age			→	PN	0.004	0.006	0.452	−0.007	0.018
BMI			→	PN	0.008	0.007	0.294	−0.007	0.023
Age			→	DR	−0.012	0.005	0.052	−0.021	−0.002
BMI			→	DR	−0.009	0.006	0.118	−0.021	0.002
Age			→	IA & P	−0.005	0.005	0.321	−0.015	0.005
BMI			→	IA & P	−0.008	0.006	0.182	−0.021	0.004
Age			→	ER & S	−0.006	0.005	0.219	−0.016	0.004
BMI			→	ER & S	−0.003	0.006	0.587	−0.016	0.008
Age			→	IL	−0.007	0.005	0.188	−0.018	0.004
BMI			→	IL	0.004	0.006	0.490	−0.008	0.017
Age			→	IIP Total	0.010	0.005	0.054	−0.004	0.024
BMI			→	IIP Total	−0.005	0.006	0.460	−0.022	0.012

Indirect effects									
EA	→	DR	→	IIP Total	0.176	0.054	0.**001**	0.081	0.305
EA	→	IA & P	→	IIP Total	0.052	0.031	0.094	−0.004	0.157
EA	→	ER & S	→	IIP Total	0.010	0.036	0.782	−0.070	0.100
EA	→	IL	→	IIP Total	0.032	0.020	0.111	−0.001	0.101
PA	→	DR	→	IIP Total	0.012	0.036	0.741	−0.055	0.113
PA	→	IA & P	→	IIP Total	−0.003	0.016	0.854	−0.065	0.050
PA	→	ER & S	→	IIP Total	−0.002	0.008	0.786	−0.031	0.015
PA	→	IL	→	IIP Total	−0.002	0.015	0.894	−0.046	0.037
SA	→	DR	→	IIP Total	−0.032	0.035	0.370	−0.136	0.053
SA	→	IA & P	→	IIP Total	−0.013	0.017	0.425	−0.083	0.015
SA	→	ER & S	→	IIP Total	0.001	0.005	0.793	−0.010	0.028
SA	→	IL	→	IIP Total	−0.016	0.016	0.305	−0.061	0.007
EN	→	DR	→	IIP Total	0.029	0.042	0.486	−0.060	0.127
EN	→	IA & P	→	IIP Total	0.016	0.020	0.424	−0.019	0.100
EN	→	ER & S	→	IIP Total	−0.001	0.005	0.793	−0.032	0.012
EN	→	IL	→	IIP Total	0.003	0.017	0.861	−0.037	0.049
PN	→	DR	→	IIP Total	0.009	0.039	0.817	−0.063	0.098
PN	→	IA & P	→	IIP Total	0.026	0.021	0.219	−0.006	0.114
PN	→	ER & S	→	IIP Total	−0.001	0.003	0.821	−0.023	0.012
PN	→	IL	→	IIP Total	0.032	0.020	0.109	0.002	0.089

*Note:* Significant results are in bold.

Abbreviations: BMI: body mass index; DR: disconnection and rejection; EA: emotional abuse; EN: emotional neglect; ER & S: excessive responsibilities and standards; IA & P: impaired autonomy and performance; IIP: inventory of interpersonal problems total score; IL: impaired limits; PA: physical abuse; PN: physical neglect; SA: sexual abuse.

## 4 Discussion

This study is the first attempt to examine the mediational role of EMSs in the relationship between childhood trauma and interpersonal problems specifically within a sample of individuals with EDs.

The results confirm that childhood trauma, EMSs, and interpersonal problems show significant variability among individuals with EDs. Additionally, childhood adversities, particularly emotional abuse, were positively associated with elevated scores across EMS domains and greater interpersonal problems, partially confirming the initial hypothesis. However, no significant associations were found for sexual abuse. Finally, the mediation analyses confirmed that EMSs, especially within the DR domain, play a key role in mediating the relationship between emotional abuse and interpersonal difficulties.

Although significant differences in BMI and age were observed across ED subtypes (e.g., higher BMI and age in BED patients), these variables alone may not fully account for the psychological and emotional distinctions. This highlights the importance of incorporating transdiagnostic factors into etiopathological models of EDs, such as those proposed by Fairburn (2008).

Regarding childhood trauma, the BED group reported higher scores than the AN‐R group on the sexual abuse subscale, a finding that aligns with studies emphasizing the link between binge eating behaviours and histories of sexual trauma (Afifi et al. 2017). Sexual abuse has been widely associated with emotional dysregulation and the development of maladaptive coping mechanisms, such as binge eating (Wonderlich et al. 2001). This suggests the potential role of trauma‐focused interventions in BED populations, which continues to be underexplored in clinical research (Brewerton 2023; Day et al. 2024).

Additionally, BN group exhibited higher scores across all schema domains compared to other groups. This indicates that patients with BN may experience more severe EMSs, which have been shown to correlate with heightened emotional instability and greater psychopathological severity. EMSs, such as those related to DR domain, likely exacerbate emotional dysregulation by reinforcing negative core beliefs about the self and others, thereby contributing to maladaptive coping mechanisms, including disordered eating behaviours (Pauwels et al. 2016). This interplay between schemas and emotional instability may underpin the higher psychopathological levels observed in BN patients, as previously documented in the literature (Meneguzzo et al. 2021). In contrast, AN‐BP and BED groups showed differences in schema domains, with AN‐BP patients presenting higher scores in domains like impaired autonomy and performance (IA&P) and excessive responsibility and standards (ER&S), supporting previous literature linking these schemas to restrictive eating behaviours (Dingemans et al. 2006). These findings are not surprising, as it is well‐documented that clinical perfectionism and high personal standards are prevalent in this population (Delaquis et al. 2024).

In terms of interpersonal functioning, the BN group reported higher total scores for interpersonal problems than both the AN‐R and BED groups, particularly in subscales measuring socially inhibited and avoidant behaviour. This finding is consistent with previous research that highlights difficulties in social functioning as a core issue for individuals with BN, potentially driven by schemas related to feelings of inadequacy and fear of rejection (Ung et al. 2017). The higher schema domains and IIP scores observed in the BN group may, in part, reflect limitations inherent to self‐report measures. Specifically, patients with BN might perceive themselves as less effective in managing interpersonal relationships, leading to inflated scores. Additionally, these findings could be influenced by state‐dependent phenomena associated with the acute phase of the disorder, rather than reflecting stable traits. Previous research has highlighted that emotional instability and interpersonal difficulties often fluctuate in response to the intensity of disordered eating behaviours (Lavender et al. 2015). Further, the BED group showed higher scores in the Intrusive/Needy domain align with studies showing that individuals with BED often seek validation and struggle with boundaries in relationships (Aloi, Rania, Sacco, et al. 2020; Brugnera et al. 2018).

The correlation analysis found significant positive associations between childhood adversities and schema domains, confirming that early trauma shapes EMSs that persist into adulthood. The most interesting results was that having experienced sexual abuse does not correlate with any schema domain. This finding can be interpreted in several ways. First, emotional and physical forms of abuse or neglect directly challenge a child's basic needs for safety, love, and belonging, leading to the development of EMSs related to mistrust, abandonment, and rejection (Young et al. 2003). In contrast, sexual abuse might be more closely linked to other psychological outcomes, such as dissociation or body‐focused concerns, rather than schema domains related to interpersonal issues like trust and autonomy. Another possible explanation regards the developmental timing: it is possible that the consequences of sexual abuse manifest lately in adulthood, through mechanisms like post‐traumatic stress disorder (PTSD) or body image issues (Bödicker et al. 2022; Grilo and Masheb 2001), which are not fully captured by schema‐focused measures. Other forms of trauma might influence earlier cognitive development, resulting in more pervasive and lasting EMSs. In fact, sexual abuse can provoke a distinct set of cognitive and emotional responses, such as shame, guilt, and body image disturbances, which may not fully align with the schema domains captured in our study, such as disconnection/rejection or impaired autonomy. Sexual trauma might lead to maladaptive coping strategies, such as self‐harm or substance abuse (Nicola et al. 2024), that are not necessarily explained by EMSs related to interpersonal problems.

The mediation analysis highlights the pivotal role of EMSs in the DR domain as mediators between childhood trauma, particularly emotional abuse, and interpersonal difficulties in individuals with EDs. The DR domain, characterized by beliefs of unworthiness, mistrust, and abandonment, has been consistently linked to maladaptive interpersonal patterns in various psychiatric conditions. For example, studies in depression and anxiety disorders have found that schemas in this domain predict heightened sensitivity to rejection and chronic relational difficulties, often perpetuating feelings of isolation and withdrawal (Harris and Curtin 2002). Similarly, in borderline personality disorder, DR schemas are implicated in the intense fear of abandonment and unstable interpersonal relationships that typify the disorder (Rosenbach and Renneberg 2014, 2015). In psychotic disorders, the DR domain has been associated with paranoia and mistrust, further underscoring its transdiagnostic relevance (Boyda et al. 2018). Finally, DR domain was the most strongly related schema domain across addictive behaviours in a recent systematic review (Vieira et al. 2023). These findings suggest that schemas within the DR domain may represent a core mechanism underlying interpersonal dysfunction across diverse psychopathologies. The centrality of the DR domain in EDs further supports its transdiagnostic importance. Individuals with heightened DR schemas may internalize relational disruptions as evidence of personal inadequacy, exacerbating both their interpersonal difficulties and the maladaptive eating behaviours used to cope with these negative beliefs. This interplay aligns with schema therapy principles, which emphasize targeting core beliefs about rejection and mistrust to interrupt cycles of maladaptive emotional and behavioural responses (Karatzias et al. 2016; Young et al. 2003). Our findings suggest that schema‐focused interventions tailored to the DR domain could be particularly effective in mitigating the impact of childhood trauma on interpersonal functioning and, by extension, ED symptoms. Addressing these schemas may also offer broader therapeutic benefits, as improving interpersonal relationships is crucial for recovery in EDs.

This study should be considered with both its strengths and limitations in mind. First, this research involved a sizable clinical sample (*N* = 321) that included males and females with a variety of eating disorders (AN‐R, AN‐BP, BN, BED). This diversity enhances the generalizability of the findings across different ED populations, unlike studies that predominantly focus on female patients with AN or BN. Further, the study applies a transdiagnostic perspective by examining core psychopathological features across different ED types that reflects the growing emphasis on shared psychological factors across EDs. Regarding the limitations, the study uses a cross‐sectional design, which limits the ability to infer causal relationships between childhood trauma, EMSs, and interpersonal problems. Longitudinal studies would provide more insight into the temporal relationships and potential causal mechanisms. Besides, the use of self‐report measures for childhood trauma could introduce recall bias or social desirability bias, potentially affecting the accuracy of the data.

## 5 Conclusion

Overall, the present findings underscore the transdiagnostic role of DR schema domain across the ED population and emphasize the significant impact of childhood trauma, particularly emotional abuse, on interpersonal functioning. Future research should explore these relationships longitudinally to better understand the developmental pathways linking childhood trauma to ED psychopathology.

## Author Contributions

**Matteo Aloi**: conceptualization; data curation; methodology; writing–original draft; writing–review and editing. **Marianna Rania**: data curation; writing–review and editing. **Elvira Anna Carbone**: data curation; writing–review and editing. **Renato de Filippis**: data curation; writing–review and editing. **Daria Quirino:** data curation. **Ettore D’Onofrio**: data curation. **Cristina Segura Garcia**: supervision; writing–review and editing.

## Ethics Statement

The study followed all relevant ethical guidelines. All procedures performed were in accordance with the ethical standards of the institutional research committee and with the 1964 Helsinki Declaration and its later amendments (or comparable ethical standards).

## Consent

Informed consent has been obtained from all participants, and the investigation has been conducted according to the best principles of research with human beings.

## Conflicts of Interest

The authors declare no conflicts of interest.

## Supporting information


**Table S1** Scatterplots of the correlation matrix among the variables of interest.

## Data Availability

The data that support the findings of this study are available from the corresponding author upon reasonable request.
