# Supplementary figures and images for: Early Maladaptive Schemas Mediate the Relationship Between Childhood Trauma and Interpersonal Problems in Eating Disorders
Source: Clin Psychol Psychother. 2025 Mar 16;32(2):e70052. doi: 10.1002/cpp.70052 (PMC11911117; doi:10.1002/cpp.70052)

**Supplementary Table 1.** Scatterplots of the correlation matrix among the variables of interest.


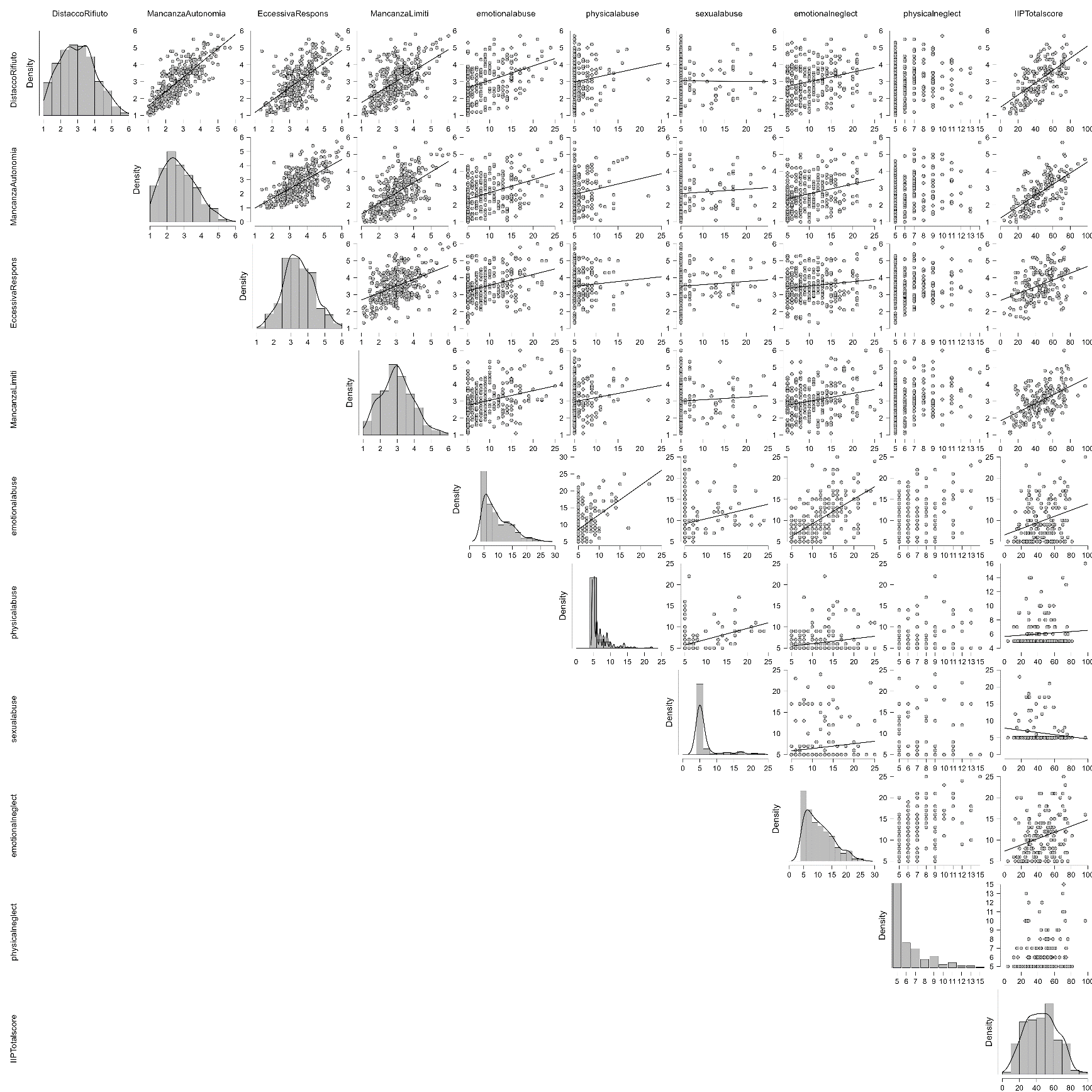

Supplement: Supplementary file 1 — Table S1 Scatterplots of the correlation matrix among the variables of interest. [file CPP-32-e70052-s001.docx]
